# Insect diversity in the Saharo-Arabian region: Revealing a little-studied fauna by DNA barcoding

**DOI:** 10.1371/journal.pone.0199965

**Published:** 2018-07-09

**Authors:** Muhammad Ashfaq, Jamal S. M. Sabir, Hosam O. El-Ansary, Kate Perez, Valerie Levesque-Beaudin, Arif M. Khan, Akhtar Rasool, Carlene Gallant, Joseph Addesi, Paul D. N. Hebert

**Affiliations:** 1 Centre for Biodiversity Genomics, University of Guelph, Guelph, ON, Canada; 2 Biotechnology Research Group, Department of Biological Sciences, King Abdulaziz University, Jeddah, Saudi Arabia; 3 Floriculture, Ornamental Horticulture and Garden Design Department, Faculty of Agriculture (El-Shatby), Alexandria University, Alexandria, Egypt; 4 Department of Plant Production, College of Food and Agriculture Sciences, King Saud University, Riyadh, Saudi Arabia; 5 Department of Biotechnology, University of Sargodha, Sargodha, Pakistan; 6 Centre for Animal Science and Fisheries, University of Swat, Mingora, Pakistan; Tierarztliche Hochschule Hannover, GERMANY

## Abstract

Although insects dominate the terrestrial fauna, sampling constraints and the poor taxonomic knowledge of many groups have limited assessments of their diversity. Passive sampling techniques and DNA-based species assignments now make it possible to overcome these barriers. For example, Malaise traps collect specimens with minimal intervention while the Barcode Index Number (BIN) system automates taxonomic assignments. The present study employs Malaise traps and DNA barcoding to extend understanding of insect diversity in one of the least known zoogeographic regions, the Saharo-Arabian. Insects were collected at four sites in three countries (Egypt, Pakistan, Saudi Arabia) by deploying Malaise traps. The collected specimens were analyzed by sequencing 658 bp of cytochrome oxidase I (DNA barcode) and assigning BINs on the Barcode of Life Data Systems. The year-long deployment of a Malaise trap in Pakistan and briefer placements at two Egyptian sites and at one in Saudi Arabia collected 53,092 specimens. They belonged to 17 insect orders with Diptera and Hymenoptera dominating the catch. Barcode sequences were recovered from 44,432 (84%) of the specimens, revealing the occurrence of 3,682 BINs belonging to 254 families. Many of these taxa were uncommon as 25% of the families and 50% of the BINs from Pakistan were only present in one sample. Family and BIN counts varied significantly through the year, but diversity indices did not. Although more than 10,000 specimens were analyzed from each nation, just 2% of BINs were shared by Pakistan and Saudi Arabia, 4% by Egypt and Pakistan, and 7% by Egypt and Saudi Arabia. The present study demonstrates how the BIN system can circumvent the barriers imposed by limited access to taxonomic specialists and by the fact that many insect species in the Saharo-Arabian region are undescribed.

## Introduction

Because insects are the major component of terrestrial metazoan biodiversity and important indicators of environmental conditions [1,2], their biomonitoring can aid efforts to conserve and restore biodiversity [3], to evaluate the impacts of climate change [4], and to protect ecological services [5,6]. However, comprehensive assessments of their diversity have been impossible because of the lack of taxonomic specialists, and because many insect species are undescribed [7]. These barriers have contributed to the current uncertainty in the global species count [8] which can only be resolved by new approaches as morphological studies could require a millennium to inventory all species [9].

Recent advances in sequencing technology have stimulated the adoption of DNA-based methods for documenting biodiversity [10,11]. The capacity of DNA barcoding to advance understanding of biodiversity at local [12–14] and continental scales [15] is now well-established. The Barcode Index Number (BIN) System [16] is a particularly important development as BINs are a strong proxy for morphological species [17,18]. Consequently, the BIN system is enabling both large-scale assessments of species diversity [19] and detailed studies of entire taxonomic assemblages [20]. The capacity of BINs to delimit species is particularly valuable in settings where prior taxonomic work has been constrained [21]. As a consequence, BINs are being used to advance biodiversity inventories [22], to reveal unknown faunas [23], and to explore biodiversity links among nations [24,25].

**Fig 1:**
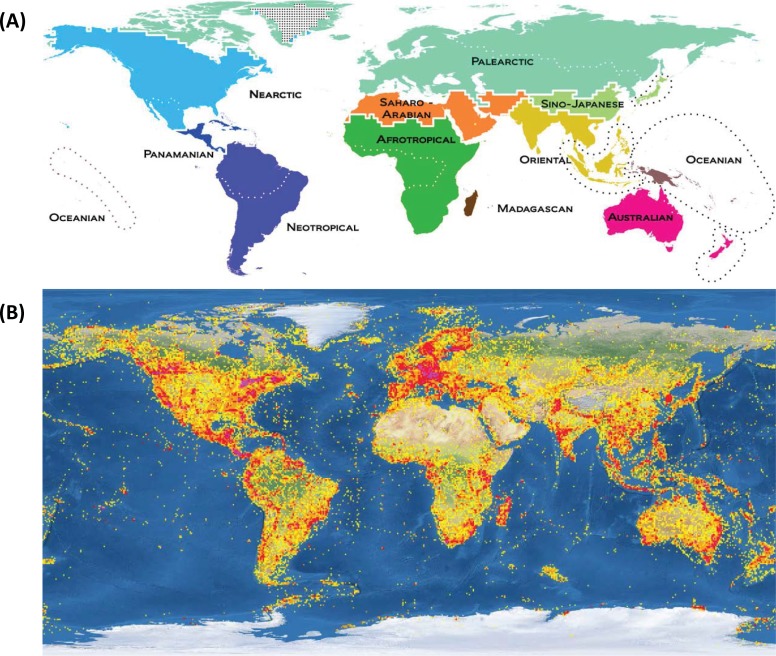
Map of the terrestrial zoogeographic regions (A; adopted from Holt et al. Science 2013; 339: 74–78) and biodiversity documentation by DNA barcoding (B; taken from the Barcode of Life Data Systems, www.boldsystems.org).

Among the 12 zoogeographic regions [26], data on insect diversity is least available for the Saharo-Arabian (Fig 1). For example, nearly half of the 371 arthropod families recently collected from the United Arab Emirates represented new records for that nation [27]. Egypt, Pakistan, and Saudi Arabia jointly comprise a land area of four million square-kilometers in the Saharo-Arabian region. Although each nation is thought to host more than 10,000 insect species, documentation of this diversity is very incomplete [28,29]. For instance, only 5,000 insect species have been reported from Pakistan [30], 3,000 from Saudi Arabia [31], and less than 2,500 from Egypt [32] in comparison with over 100,000 from Canada and the USA. The limited availability of biodiversity data for the Saharo-Arabian region restricts the recognition of invasive taxa and threatened species while also constraining deeper assessments of patterns of community structure and faunal evolution.

Aside from a lack of taxonomic resources, inaccessible terrain and political instability have contributed to the poor documentation of Saharo-Arabian biodiversity. This knowledge gap can be addressed by coupling efficient sampling methods, such as Malaise trap [33] with subsequent DNA barcode analysis to speed determinations of alpha and beta diversity and overlap [19]. By using this approach to examine patterns of insect diversity in Egypt, Pakistan, and Saudi Arabia, the present study provides a model for biodiversity surveillance in regions with limited previous biodiversity research.

## Materials and methods

### Ethics statement

No specific permissions were required for this study. The study did not involve endangered or protected species.

### Insect collection

No single collection method allows a comprehensive assessment of insect diversity [34,35]. As a result, diverse sampling methods are employed. These methods include, but are not limited to, light traps, Malaise traps, pitfall traps, pyrethrum knockdown, and Tullgren extractors. Although Malaise traps are not an all-purpose collection method [36], they are generally accepted as the most cost- and time-effective [37], leading to their frequent use in biodiversity assessments [38]. Reflecting this fact, the Global Malaise Trap Program (http://globalmalaise.org/) has adopted this sampling method, and coupled it with DNA barcoding to advance understanding of insect diversity. As one element of this program, the present study deployed Malaise traps at four locations in three countries. One trap was installed at the Pakistan Museum of Natural History, Islamabad (33.686° N, 73.076° E) in the Shakarparian forest. Insects were collected from 7 February–13 December 2012 excepting 10 days in August/September and one week in July when samples were lost due to storm damage producing 39 collection events (weeks). A second trap was installed at Hada AL-Sham, a green valley, near Makkah, Saudi Arabia (21.795° N, 39.711° E) from 9 April–3 July 2014 (13 weeks) and from November 2014–February 2015 (5 weeks). The other traps were installed at Mostafa Kamel Village (30.92° N, 29.76° E) and Antoniodes Gardens (31.204° N, 29.95° E), Egypt. Both the sampling sites are situated on the delta of river Nile. Weekly samples were collected at site 1 from 27 May–7 October 2013 (20 weeks) and at site 2 from 29 May–18 September 2013 (17 weeks). Insects were collected into 95% ethanol, and samples were stored at -20°C until shipment to the Centre for Biodiversity Genomics at Guelph for DNA barcode analysis.

### Molecular analysis

Specimens were sorted to order, arrayed, labeled, databased, and tissue sampled following standard workflows. Small specimens were transferred directly to 96-well microplates, and vouchers were recovered after DNA extraction for imaging and curation [39]. DNA extraction, PCR amplification, and sequencing were performed at the Canadian Centre for DNA Barcoding following standard protocols (http://ccdb.ca/resources). Except for hemipterans, PCR amplification of COI-5′ was performed with primers C_LepFolF and C_LepFolR (http://ccdb.ca/site/wp-content/uploads/2016/09/CCDB_PrimerSets.pdf) following PCR conditions; 94°C (1 min), 5 cycles at 94°C (40 s), 45°C (40 s), 72°C (1 min); 35 cycles at 94°C (40 s), 51°C (40 s), 72°C (1 min) and a final extension at 72°C (5 min). These primers are mixtures of LepF1 [40] /LCO1490 [41] and LepR1 [40] /HCO2198 [41], respectively. PCR amplification of hemipterans was conducted with LepF2_t1 [42] and LepR1 using the same thermocycling regime as above. All amplification reactions included 10.5 μL (or 5.25 μL) of standard PCR ingredients [40] and 2 μL (or 1 μL) of DNA template. PCR products were analyzed using the E-gel 96 system (Invitrogen Inc.) and amplicons were sequenced using BigDye v3.1 (Applied Biosystems) on an ABI 3730XL. Sequences were assembled, aligned, and edited using CodonCode Aligner (CodonCode Corporation, USA) and submitted to Barcode of Life Data Systems (BOLD) (www.boldsystems.org) [43]. All sequences generated in this study are accessible on BOLD under the dataset DS-MAREG (dx.doi.org/10.5883/DS-MAREG).

### Data analysis

Sequences meeting quality criteria (>507 bp, <1% Ns, no stop codon or contamination flag) were assigned to a BIN by the Refined Single Linkage (RESL) algorithm on BOLD [16] which runs monthly on all eligible sequences. Shorter sequences (<507 bp), meeting all the other criteria, were assigned to the matching pre-existing BINs containing longer sequences. Results of this analysis are accessible through individual BIN pages. With few exceptions, each specimen was assigned to a family by sequence matches or by morphological analysis to existing records on BOLD (http://www.boldsystems.org/index.php/IDS_OpenIdEngine). The family level assignment of specimens was based on 90% or higher match of the unknown sequence with the known sequence. Sequences and their associated taxonomic data were subsequently downloaded from BOLD for analysis. Diversity indices, BIN/family incidences over time, and weather-diversity relationships were only examined for Pakistan since this was the sole location with collections for >75% of the year. Monthly mean temperature and relative humidity (RH) values for this site were obtained from the Pakistan Meteorological Department, Islamabad. Diversity indices, Simpson’s D [44] and Shannon’s H [45] were calculated for each collection event. The significance of diversity variation among the collection events was determined with a χ^2^ test. BIN overlap among sites was calculated in Excel while BIN accumulation curves were generated on BOLD.

## Results

The four Malaise traps collected 53,092 insects including 22,624 from Pakistan, 18,391 from Egypt (6,854 at site 1, 11,537 at site 2), and 12,077 from Saudi Arabia. Although these specimens were assigned to 17 orders, most were Diptera (42%), Hymenoptera (29%), Hemiptera (13%), Lepidoptera (7%) or Coleoptera (4%). A χ^2^ test showed that the proportion of each order varied among the collection sites (*P* <0.05) (Table 1). Although 84% (44,432) of the specimens generated barcodes, sequence recovery varied among sites with the lowest recovery from Saudi Arabia (79%). Because temperatures in Makkah often exceeded 40°C during the collection period, preservation was likely compromised [46]. Variation in barcode recovery was observed among the major orders at each site, although these differences varied among sites (χ^2^ = 54.98, *P* <0.05) (Table 1). Considering all sites, success in barcode recovery was higher for Lepidoptera (97%) and Diptera (92%) than for Coleoptera (70%) and Hemiptera/Hymenoptera (75%/76%).

**Table 1:** Insect diversity analysis by Malaise trap collections at four sites in three countries.

	Egypt			Pakistan	Saudi Arabia	Total
	Site 1	Site 2	Egypt total			
Collection period	27 May– 7 Oct, 2013	29 May– 18 Sep, 2013		15 Feb– 6 Dec 2012	9 Apr– 3 July, 2014 + one week each in Nov, 2014 –Feb, 2015	
Total catch (specimens)	6854	11537	18391	22624	12077	53092
DNA barcodes recovered (%)	5611 (82)	10169 (88)	15780 (86)	19068 (84)	9584 (79)	44432 (84)
BINs	571	636	991	2248	728	3682
Singleton BINs	256	239	367	1052	323	1566
Orders	14	12	14	17	12	17
Families	129	137	164	214	132	254
Most common orders: n (% of total catch):						
i) Coleoptera	324 (5)	153 (1)	477 (3)	1083 (5)	702 (6)	2262 (4)
ii) Diptera	4717 (69)	3280 (28)	7997 (43)	11799 (52)	2559 (21)	22355 (42)
iii) Hemiptera	197 (3)	1145 (10)	1342 (7)	1592 (7)	4097 (34)	7031 (13)
iv) Hymenoptera	981 (14)	5008 (43)	5989 (33)	6684 (30)	2847 (24)	15520 (29)
v) Lepidoptera	389 (6)	1583 (14)	1972 (11)	1028 (5)	622 (5)	3622 (7)
Chi-square = 124.8; *P*<0.05						
DNA barcodes recovered: n (%)						
i) Coleoptera	254 (78)	137 (90)	391 (82)	877 (81)	326 (46)	1594 (70)
ii) Diptera	3898 (83)	3161 (96)	7059 (89)	11126 (94)	2318 (91)	20503 (92)
iii) Hemiptera	125 (63)	833 (73)	958 (71)	1026 (64)	3298 (80)	5282 (75)
iv) Hymenoptera	780 (80)	4138 (83)	4918 (82)	4728 (71)	2139 (75)	11785 (76)
v) Lepidoptera	366 (94)	1568 (99)	1934 (98)	970 (94)	609 (98)	3513 (97)
Chi-square = 54.98; *P*<0.05						
Order diversity: BINs (BIN: specimen ratio)						
i) Coleoptera	58 (0.23)	37 (0.27)	81 (0.21)	207 (0.24)	76 (0.23)	348 (0.22)
ii) Diptera	230 (0.06)	261 (0.08)	381 (0.05)	818 (0.07)	214 (0.09)	1285 (0.06)
iii) Hemiptera	30 (0.24)	52 (0.06)	71 (0.07)	161 (0.16)	81 (0.02)	277 (0.05)
iv) Hymenoptera	191 (0.24)	195 (0.05)	327 (0.07)	829 (0.18)	244 (0.11)	1328 (0.11)
v) Lepidoptera	41 (0.11)	56 (0.04)	83 (0.04)	155 (0.16)	65 (0.11)	281 (0.08)
Chi-square = 0.35; *P*>0.05						

By considering sequence matches to records on BOLD, the specimens with barcodes were assigned to 254 families. Most of these families (216/254) belonged to five orders: Diptera (61), Hymenoptera (44), Coleoptera (42), Lepidoptera (38), and Hemiptera (31). The samples from Pakistan included 214 families while 132 were collected in Saudi Arabia and 164 in Egypt (129 at site 1 and 137 at site 2).

The 42,510 qualifying sequences were assigned to 3,682 BINs with 2,248 (61%) derived from Pakistan, 728 (20%) from Saudi Arabia, and 991 (27%) from Egypt (571 = 16% at site 1; 636 = 17% at site 2). 1,601 sequences missed the BIN assignment due to short reads (<507 b) while 101 due to high number of ambiguous bases (>1%). Another 220 sequences could not be assigned to a BIN, though they met the quality criteria. Moreover, more than half (1192/1,922) of the sequences that failed a BIN assignment could be associated with a known BIN through NJ clustering. Nearly half (43%) of all BINs (1,566/3,682) were represented by a single specimen.

**Fig 2:**
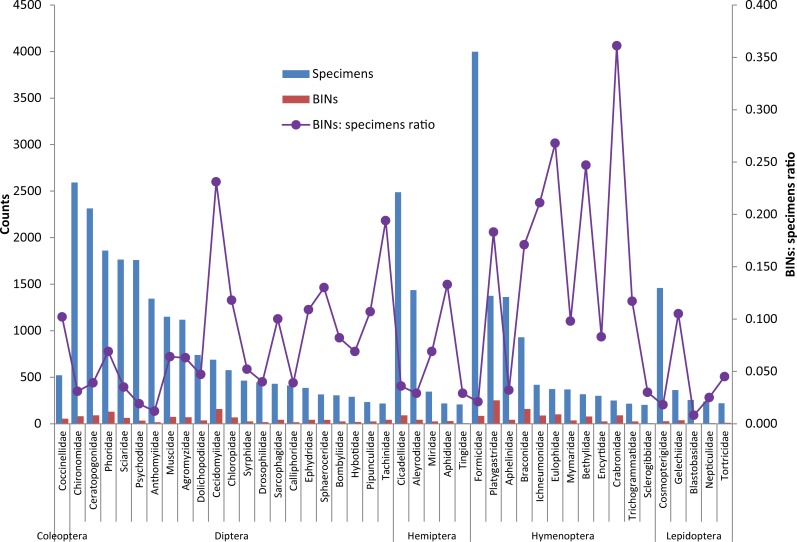
Abundance of families with their corresponding BINs (bars) and the BIN/specimen ratio (line). Only families with >100 specimens were analyzed.

The BIN/specimen ratio (0.06 vs 0.23) was lower for the most frequent order, Diptera (*n* = 22,355) than for the least frequent, Coleoptera (*n* = 2,262) (Table 1). This pattern was also evident at a family level as the three commonest families had lower BIN/specimen ratios than many of the infrequently collected families. For example, the Formicidae (*n* = 3,997, ratio = 0.02), Chironomidae (*n* = 2,593, ratio = 0.03) and Cicadellidae (*n* = 2,488, ratio = 0.04) had much lower ratios than the uncommon Crabronidae (*n* = 249, ratio = 0.36), Eulophidae (*n* = 373, ratio = 0.27) and Bethylidae (*n* = 316, ratio = 0.25) (Fig 2).

Among the 254 insect families, Formicidae was the most abundant (*n* = 3997) while the Crabronidae was the most diverse (BIN: specimen ratio = 0.36). While Formicidae dominated both (*n* = 1896) Egyptian sites, Chironomidae (*n* = 2422) was most common in Pakistan, and Cicadellidae (*n* = 2146) in Saudi Arabia. Eulophidae had the highest BIN: specimen ratio (0.27) in Egypt, while Cecidomyiidae had the highest (0.35) in Pakistan, and Platygastridae (0.27) in Saudi Arabia.

### Diversity analysis at the Pakistan site

**Fig 3:**
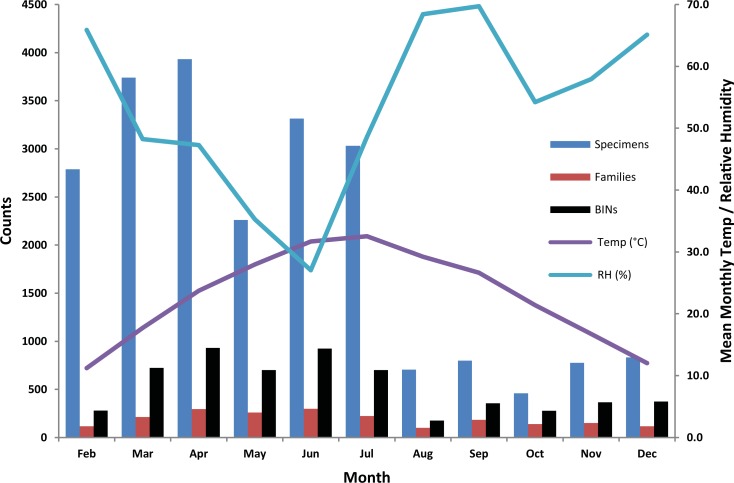
Number of insects, families, and BINs (bars) collected by the Malaise trap deployed in Islamabad, Pakistan versus temperature and relative humidity data (lines).

The 19,068 specimens from Pakistan with barcodes included representatives of 15 orders, 214 families, and 2,248 BINs. Specimens of Diptera (11,126), Hymenoptera (4,728), Hemiptera (1,026), Lepidoptera (970), and Coleoptera (877) dominated the collections with only two other orders contributing more than 100 records (Orthoptera and Psocodea were each represented by six families and by 29/16 BINs respectively). More specimens (16,031 versus 6,593) and BINs (1,592 versus 1,155) were collected from February–June than from July–December, a difference associated with the higher temperatures and lower humidity in the first half of the year (Fig 3).

**Fig 4:**
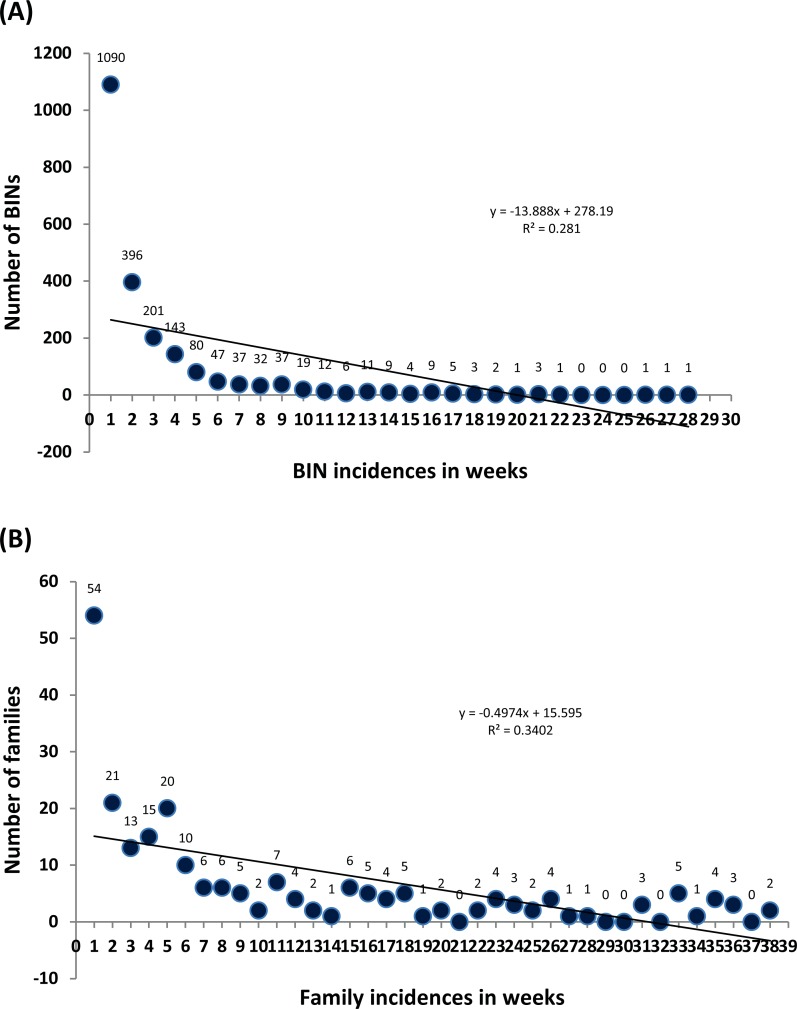
Incidence of BINs (A) and families (B) in the 39 collections from Islamabad, from February to December 2012.

Most families (54) and BINs (1,090) were encountered only once in the 39 collection events, but two families (Agromyzidae, Formicidae) and one BIN (ACF1938 –Formicidae) were detected in most weeks (38 and 28, respectively) (Fig 4A and 4B). Just 27 BINs were represented by >100 records and only 26 occurred in more than a third of the collection events (S1A and S1B Fig). The number of families and BINs varied significantly (P<0.01) among collection events (Table 2). Diversity indices for family and BIN richness in each collection event were determined by Simpson’s D and Shannon’s H and compared among collection events (Table 2). Simpson’s D for families ranged from 0.20–0.97 and for BINs from 0.50–0.99, while Shannon’s H for families ranged from 0.63–3.70 and for BINs from 1.50–5.10. A contingency χ^2^ test indicated there was no significant variation in family/BIN diversity among collection events.

**Table 2:** Number of insect specimens, barcodes, families and BINs recovered from weekly Malaise trap collections.

Month	Week	Specimens	Barcodes	Number of Families (D / H)	Number of BINs (D / H)
February	1	1495	1454	35 (0.2 / 0.63)	91 (0.51 / 1.48)
	2	1045	1027	35 (0.44 / 1.2)	105 (0.72 / 2.23)
	3	248	228	41 (0.89 / 2.89)	86 (0.94 / 3.69)
March	4	534	508	34 (0.86 / 2.4)	105 (0.94 / 3.47)
	5	851	819	34 (0.72 / 1.87)	113 (0.84 / 2.74)
	6	1441	1363	70 (0.91 / 2.97)	280 (0.94 / 4.13)
	7	914	843	71 (0.94 / 3.24)	237(0.97 / 4.48)
April	8	757	698	61 (0.93 / 3.18)	206 (0.97 / 4.38)
	9	1541	1323	87 (0.94 / 3.34)	306 (0.97 / 4.5)
	10	1035	863	87 (0.95 / 3.47)	258 (0.98 / 4.72)
	11	598	546	61 (0.92 / 3.05)	184 (0.97 / 4.35)
May	12	681	615	66 (0.92 / 3.0)	193 (0.96 / 4.18)
	13	718	549	78 (0.95 / 3.5)	232 (0.99 / 4.91)
	14	488	401	54 (0.88 / 2.87)	136 (0.91 / 3.71)
	15	372	322	62 (0.95 / 3.44)	161 (0.99 / 4.67)
June	16	1027	799	80 (0.93 / 3.44)	285 (0.91 / 4.95)
	17	267	200	50 (0.96 / 3.50)	116 (0.99 / 4.46)
	18	627	501	71 (0.95 / 3.54)	224 (0.99 / 5.0)
	19	1392	981	94 (0.95 / 3.53)	356 (0.99 / 5.11)
July	20	505	324	58 (0.95 / 3.46)	168 (0.99 / 4.71)
	21	2063	1543	105 (0.89 / 3.2)	409 (0.96 / 4.8)
	22	454	349	59 (0.93 / 3.28)	158 (0.98 / 4.53)
August	23	415	200	57 (0.97 / 3.66)	112 (0.99 / 4.41)
	24	138	61	23 (0.83 / 2.41)	37 (0.96 / 3.24)
	25	153	93	25 (0.73 / 1.91)	39(0.93 / 3.03)
September	26	128	103	24 (0.78 / 2.22)	41 (0.9 / 2.95)
	27	281	178	57 (0.93 / 3.43)	117 (0.99 / 4.47)
	28	190	163	51 (0.96 / 3.49)	94 (0.97 / 4.11)
	29	199	154	48 (0.97 / 3.56)	112 (0.99 / 4.51)
October	30	192	166	47 (0.97 / 3.06)	91 (0.99 / 4.29)
	31	62	57	30 (0.94 / 3.05)	45 (0.99 / 3.72)
	32	52	44	21 (0.95 / 2.85)	36 (0.99 / 3.5)
	33	153	135	41 (0.96 / 3.35)	110 (0.99 / 4.38)
November	34	83	78	30 (0.91 / 2.95)	53 (0.98 / 3.75)
	35	471	422	60 (0.95 / 3.42)	203 (0.99 / 4.79)
	36	14	13	9 (0.86 / 1.84)	9 (0.94 / 2.09)
	37	208	184	42 (0.96 / 3.35)	115 (0.99 / 4.46)
December	38	431	395	54 (0.95 / 3.32)	194 (0.99 / 4.79)
	39	401	366	59 (0.96 / 3.43)	190 (0.99 / 4.79)
Chi-square		*P* < 0.01	*P* < 0.01	*P* < 0.01 (*P* >1 / *P* > 1)	*P* < 0.01 (*P* >1 / *P* > 1)

D = Simpson’s diversity index (Simpson 1949); H = Shannon’s diversity index (Shannon and Weaver 1948).

### BIN overlap among collection sites

**Fig 5:**
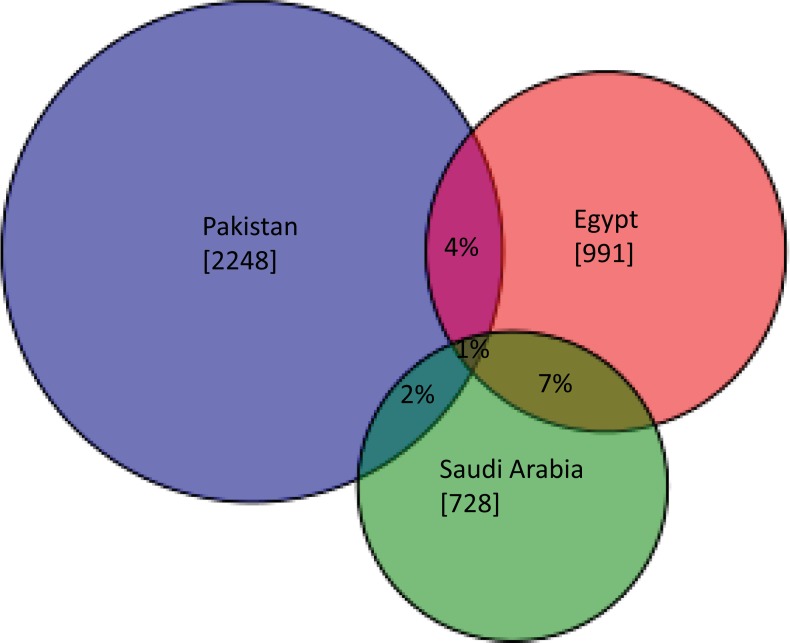
BIN overlap among the three countries–Egypt, Pakistan, Saudi Arabia.

**Fig 6:**
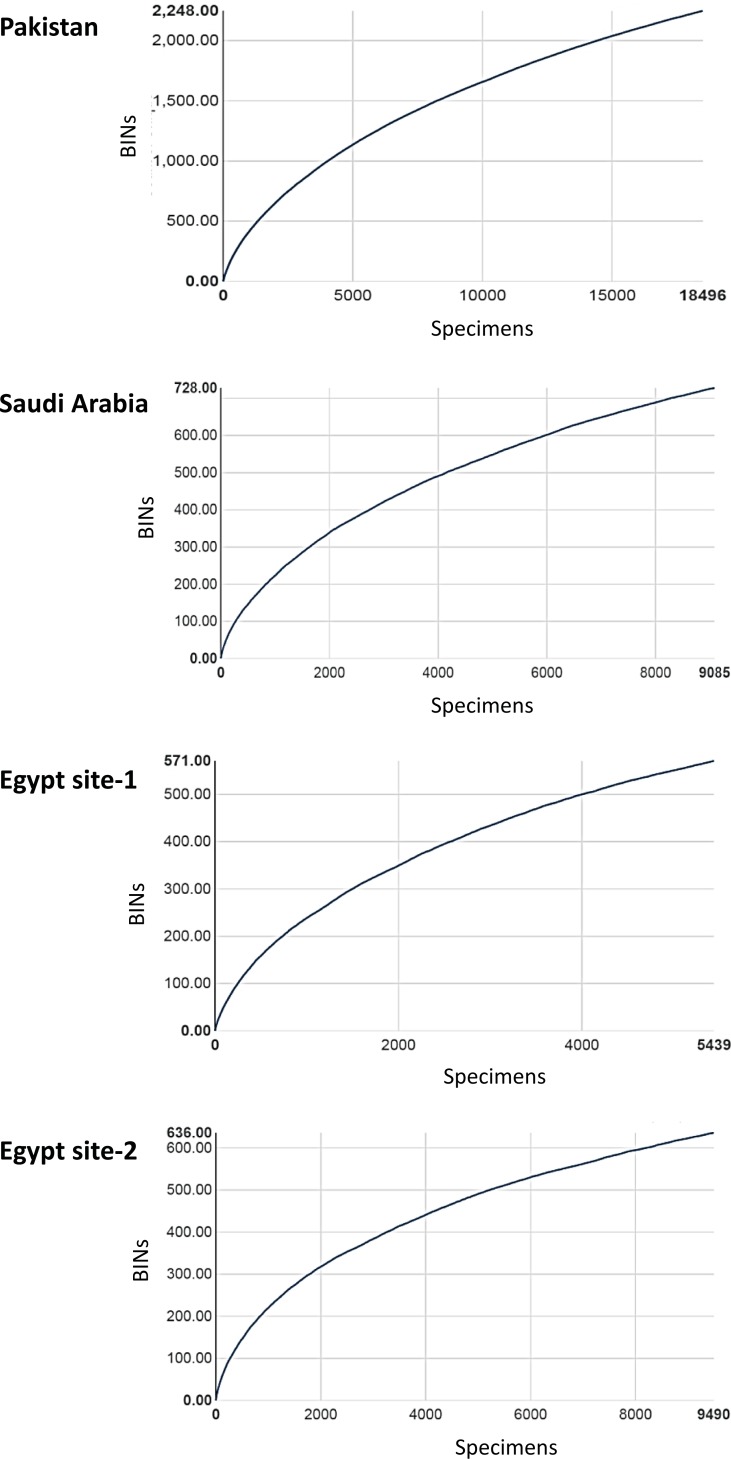
Accumulation curve for Barcode Index Numbers (BINs) recovered from insect samples collected at the four Malaise trap sites.

BIN assignments allowed quantification of the overlap in species assemblages among the four sites. Among the 2,248 BINs collected in Pakistan and the 728 in Saudi Arabia, just 2% were shared (Fig 5). The overlap between Egypt (991 BINs from the two trap sites) and Saudi Arabia was 7% while that between Egypt and Pakistan was only 4%. The BIN overlap between the two Egyptian sites was higher (19%), but the sites were just 45 km apart. The BIN accumulation curves showed no sign of reaching an asymptote at any of the sites (Fig 6).

## Discussion

Comprehensive assessments of the insect species present at even a single locale have, until now, been impossible. For example, the analysis of 129,494 insects from a single 0.5 hectare site in Panama required contributions from 102 taxonomists over an 8-year interval to place them to 6,144 putative species [47]. Despite this massive effort, just 23% of the specimens gained a formal species assignment, and nearly half of all specimens had to be excluded from the identification effort because they belonged to groups lacking taxonomic specialists. Viewed from this perspective, the capacity of a single Malaise trap to gather 30,000–50,000 specimens per year represents an overwhelming challenge for any identification effort employing morphological approaches. The present study overcame this barrier by using DNA barcoding and the BIN system to assign each specimen to a putative species. Although prior studies have demonstrated the power of this approach for work on well-known faunas [19,48,49], the present study breaks new ground by employing this method to explore insect diversity in a region with limited biodiversity knowledge.

This study examined 53,000 specimens derived from single traps deployed for 17–39 weeks at sites in Egypt, Pakistan, and Saudi Arabia. As these deployments totaled 93 weeks, trap catches averaged 570 specimens a week. As reported in prior studies [19,48,50], Diptera and Hymenoptera dominated the catch. BIN compliant sequences were recovered from 84% of the specimens, a value similar to those reported for specimens from Malaise traps deployed in Canada [19] and Europe [48]. Recovery did vary among insect orders, being higher for Diptera and Lepidoptera than for Coleoptera, Hemiptera and Hymenoptera, a pattern documented in prior studies that likely reflects primer mismatches [19]. Targeting shorter sequence fragments or employing HTS approaches [51] may alleviate the issues related to primer mismatches.

The abundance and diversity of collected insects varied over the collection period. The pattern of bigger and diverse catches in earlier than the later months of the year coincided with a rise in temperature from March to June and a fall from September to December. Weather is known to influence both spatial and temporal patterns of insect communities [52]. It is also known that insect emergence is driven by temperature that also affects their development, survival and abundance [53].

Analysis indicated that the 44,000 specimens which generated sequences belonged to 3,682 BINs with representatives of 254 families and 17 insect orders. These samples could not have been processed morphologically because such a large fraction of the Saharo-Arabian insect fauna is undescribed. For example, 20% of the species encountered in a recent survey of the United Arab Emirates were new, even though the groups analyzed were among those with the best taxonomy [27]. Because of this taxonomic barrier, the species present in Malaise trap samples from the Saharo-Arabian region have never been comprehensively assessed. Despite this lack of directly comparable data, the present study has shown that the barcode analysis of specimens from brief sampling of a few sites recovered half as many insect species as reported from all prior studies in these nations. The present results further indicated that more sampling is required to ascertain the number of species in each nation and the extent of overlap in their faunas. Certainly, the BIN overlap values (2–7%) reported in this study are underestimates of actual faunal overlap because of under-sampling. Because measures of endemism [54] play such an important role in conservation planning [55,56], further surveys of insect diversity in the Saharo-Arabian region are needed. These surveys can potentially employ several other methods of passive collection such as emergence traps, light traps, pitfall traps and coloured pan traps. The lower level of sequence recovery observed in samples from one of the study locations could be related to poor preservation or storage condition of specimens [46]. The issue of deteriorating DNA quality in preserved samples may be resolved by using better preservatives, such as 95% ethanol [57]. While the sampling effort required to properly estimate overall diversity and overlap values cannot be determined without further sampling, the analysis of a Malaise trap catch from each ecoregion within these nations would represent an important first step.

This present study has demonstrated how the BIN system can circumvent the barriers imposed by limited access to taxonomic specialists and by the fact that many insect species in the Saharo-Arabian region are undescribed. As such, it demonstrates how biodiversity analysis can be accelerated in regions that have seen little exploration [58]. Because it remains critical to extend the DNA barcode reference library through specimen-based analysis [59], it is important that new sequencing platforms are leading to substantial reductions in analytical costs [51]. Moreover, once libraries are well parameterized, metabarcoding studies [50,60] will permit detailed tracking of biodiversity trajectories at scales that would otherwise be impossible.

## Supporting information

S1 Fig Spatial (A) and temporal (B) abundance of BINs. (A) shows BINs with at least 100 specimens in the total collection while (B) shows BINs detected in at least 14 of 39 collection events.
(PDF)
