# Supplementary figures and images for: Insect diversity in the Saharo-Arabian region: Revealing a little-studied fauna by DNA barcoding
Source: PLoS One. 2018 Jul 9;13(7):e0199965. doi: 10.1371/journal.pone.0199965 (PMC6037371; doi:10.1371/journal.pone.0199965)

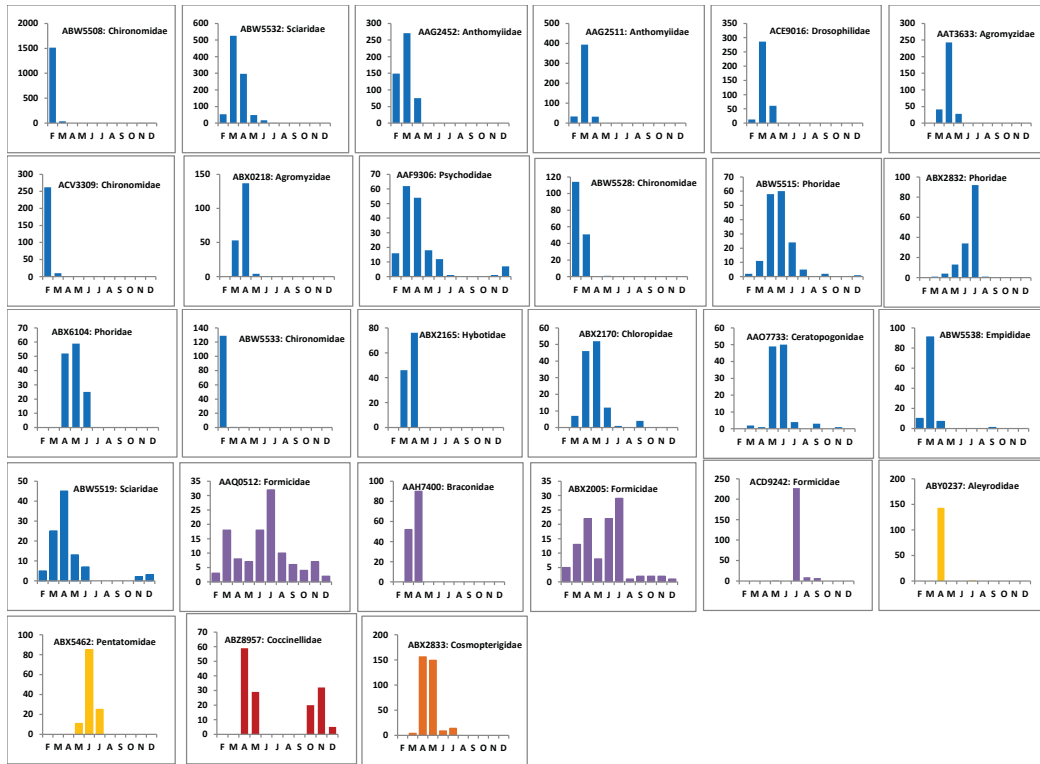

(A)

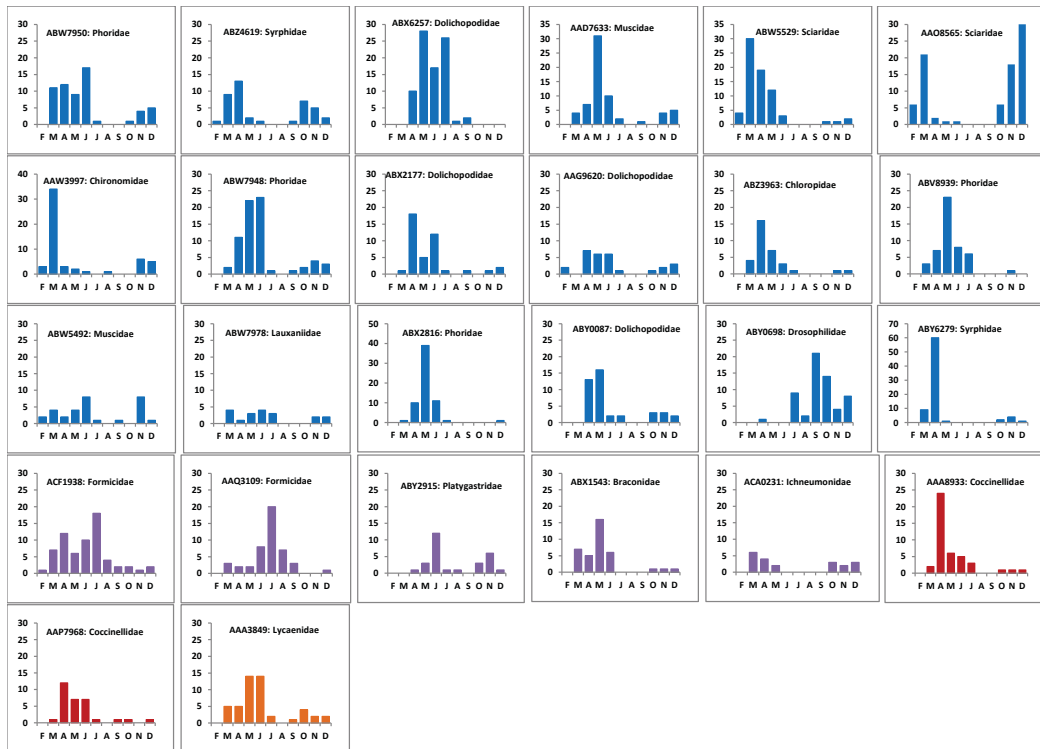

(B)

Fig. S3

Supplement: S1 Fig — Spatial (A) and temporal (B) abundance of BINs. (A) shows BINs with at least 100 specimens in the total collection while (B) shows BINs detected in at least 14 of 39 collection events. (PDF) [file pone.0199965.s001.pdf]
